# Effect of temperature on the unimodal size scaling of phytoplankton growth

**DOI:** 10.1038/s41598-020-79616-0

**Published:** 2021-01-13

**Authors:** Cristina Fernández-González, Emilio Marañón

**Affiliations:** 1 Department of Ecology and Animal Biology, Universidade de Vigo, Vigo, Spain; 2 Centro de Investigación Mariña (CIM-UVigo), Vigo, Spain

**Keywords:** Ecology, Ocean sciences

## Abstract

Contrary to predictions by the allometric theory, there is evidence that phytoplankton growth rates peak at intermediate cell sizes. However, it is still unknown if this pattern may result from the effect of experimental temperature. Here we test whether temperature affects the unimodal size scaling pattern of phytoplankton growth by (1) growing *Synechococcus* sp., *Ostreococcus tauri*, *Micromonas commoda* and *Pavlova lutheri* at 18 °C and 25 °C, and (2) using thermal response curves available in the literature to estimate the growth rate at 25 °C as well as the maximum growth rate at optimal temperature for 22 species assayed previously at 18 °C. We also assess the sensitivity of growth rate estimates to the metric employed for measuring standing stocks, by calculating growth rates based on in vivo fluorescence, chlorophyll *a* concentration, cell abundance and biomass (particulate organic carbon and nitrogen content). Our results show that the unimodal size scaling pattern of phytoplankton growth, with a peak at intermediate cell sizes, is observed at 18 °C, 25 °C and at the optimal temperature for growth, and that it prevails irrespective of the standing-stock metric used. The unimodal size scaling pattern of phytoplankton growth is supported by two independent field observations reported in the literature: (i) a positive relationship between cell size and metabolic rate in the picophytoplankton size range and (ii) the dominance of intermediate-size cells in nutrient-rich waters during blooms.

## Introduction

Phytoplankton size structure is one of the main factors that control the trophic organisation of planktonic communities^1,2^. Small cells (< 5 µm in equivalent spherical diameter, ESD) have a high affinity for nutrients and low resource requirements, which makes them particularly well-adapted to oligotrophic regions, where they support complex food webs that favour nutrient recycling and sustain low carbon export to upper trophic levels. In contrast, larger cells (≥ 5–10 µm in ESD) dominate in nutrient-rich environments, characterised by shorter and simpler microbial food webs that allow higher carbon transfer towards upper trophic levels and the ocean’s interior^3^.

The metabolic theory of ecology (MTE) predicts that temperature and size are two universal factors that control the metabolic rate of all organisms. According to this theory, individual metabolic rates (R) scale with body size (expressed as mass, M) to the power of ¾, ($R\propto {M}^\frac{3}{4}$, Kleiber’s rule)^4–6^, which means that mass-specific metabolic rates (metabolic rate per unit of mass, R^M^) and growth rates increase with decreasing body size following a size-scaling exponent of − ¼ ($R^{M} \propto M^{{ - \frac{1}{4}}}$).

It is still unclear if Kleiber’s rule applies to unicellular organisms. Raven^7^ found that the expected, continued increase in growth rates with decreasing cell size was not observed for cyanobacteria (cell volume range: 0.1–50 µm^3^; 0.5–4.5 µm in ESD) and chlorophytes (1–1000 µm^3^; 1–12 µm). Instead, maximum growth rates started to decrease below 5 µm^3^ for cyanobacteria and below 50 µm^3^ for chlorophytes, as predicted on the basis of the increased fraction of cell volume occupied by non-scalable components. This unimodal relationship between cell size and growth rate was later corroborated by field observations. An experiment with pico- and nanophytoplankton natural assemblages, covering a cell size range from 0.6 to 10 µm in ESD, found that growth rates peaked at cell diameters between 2 and 3 µm, regardless of nutrient conditions or water temperature^8^. A further compilation of phytoplankton growth rates, obtained with the dilution technique or derived from ^14^C-based primary production data, showed that the relationship between mass-specific growth rate and cell size, covering from prokaryotes to large eukaryotes (0.6–10.5 µm in ESD), was unimodal and peaked at approximately 5.4 and 2.8 µm of ESD for rates estimated with the dilution technique and ^14^C, respectively^9^.

In a laboratory study of 22 species spanning seven orders of magnitude in cell volume and belonging to five phyla, Marañón et al.^10^ found a unimodal pattern with the highest growth rates represented by cell sizes between 30 and 300 µm^3^ in cell volume (equivalent to 4–8 µm in ESD). This study, and subsequent Droop-based modelling work by Ward et al.^11^, suggested that the unimodal size scaling pattern arises from a trade-off between size-dependent nutrient uptake, requirement and assimilation. Small cells would have their maximum growth rate limited by a low nutrient uptake relative to their requirements and by the increasing fraction of cell volume occupied by non-scalable components^7^, whereas large cells would be penalized by the larger intracellular distances to transport nutrients from the uptake to the metabolic processing sites and by the package effect, which reduces light absorption efficiency^10^, both ultimately leading to decreased nutrient assimilation^2^.

The cultures used by Marañón et al.^10^ were all grown at 18 °C. Although this approach is ecologically relevant to ascertain competitive dynamics in situ because all populations in an assemblage experience the same temperature, the species used were phylogenetically distinct and had been isolated from different locations in the ocean. This could result in different responses to temperature as well as different optimal temperatures for growth^12^. For instance, some of the strains used in that study, such as those of the cyanobacteria *Prochlorococcus* and *Synechococcus*, had been isolated in tropical regions, which means that their optimal growth temperatures were likely higher than 18 °C. The possibility exists, therefore, that the decrease in growth rates with decreasing cell size observed in the pico- to small nano-phytoplankton size range may have resulted from a suboptimal growth temperature. In this regard, Sal et al.^13^, after correcting for phylogenetic differences between species of different cell sizes, concluded that the observed curvature in the size-scaling of growth rate reflects the adaptation of picophytoplankton to the warm conditions typically found in oligotrophic environments.

Here we test the hypothesis that the unimodal size scaling of phytoplankton growth reported by Marañón et al.^10^ arises from an effect of temperature. With this aim, we use two approaches. First, to examine the extent to which a temperature of 18 °C limits the growth rate of the smallest organisms, belonging to the left-hand side of the unimodal pattern, we grew four species, from 0.41 to 45 µm^3^ in cell volume, at two different temperatures (18 °C and 25 °C), and determined their growth rate using different metrics for assessing standing stocks. Secondly, we used a large data set of phytoplankton temperature-growth curves^14–17^ to obtain empirical functions that relate temperature to growth rate in the same species used by Marañón et al.^10^. This allowed us to infer an estimated maximum growth rate at 25 °C, as well as to obtain the growth rate-cell size relationship at the optimal temperature for each species. These two approaches combined serve to determine if the unimodal size-scaling pattern observed at 18 °C persists at 25 °C and at the optimal temperature for each species.

## Results

We used laboratory experiments combined with database analyses to investigate the relationship between cell size and growth rate of phytoplankton at 18 °C, 25 °C and the optimal temperature for growth. Our results suggest that the unimodal size scaling of phytoplankton growth persists at all these temperatures.

### Laboratory experiments

**Figure 1:**
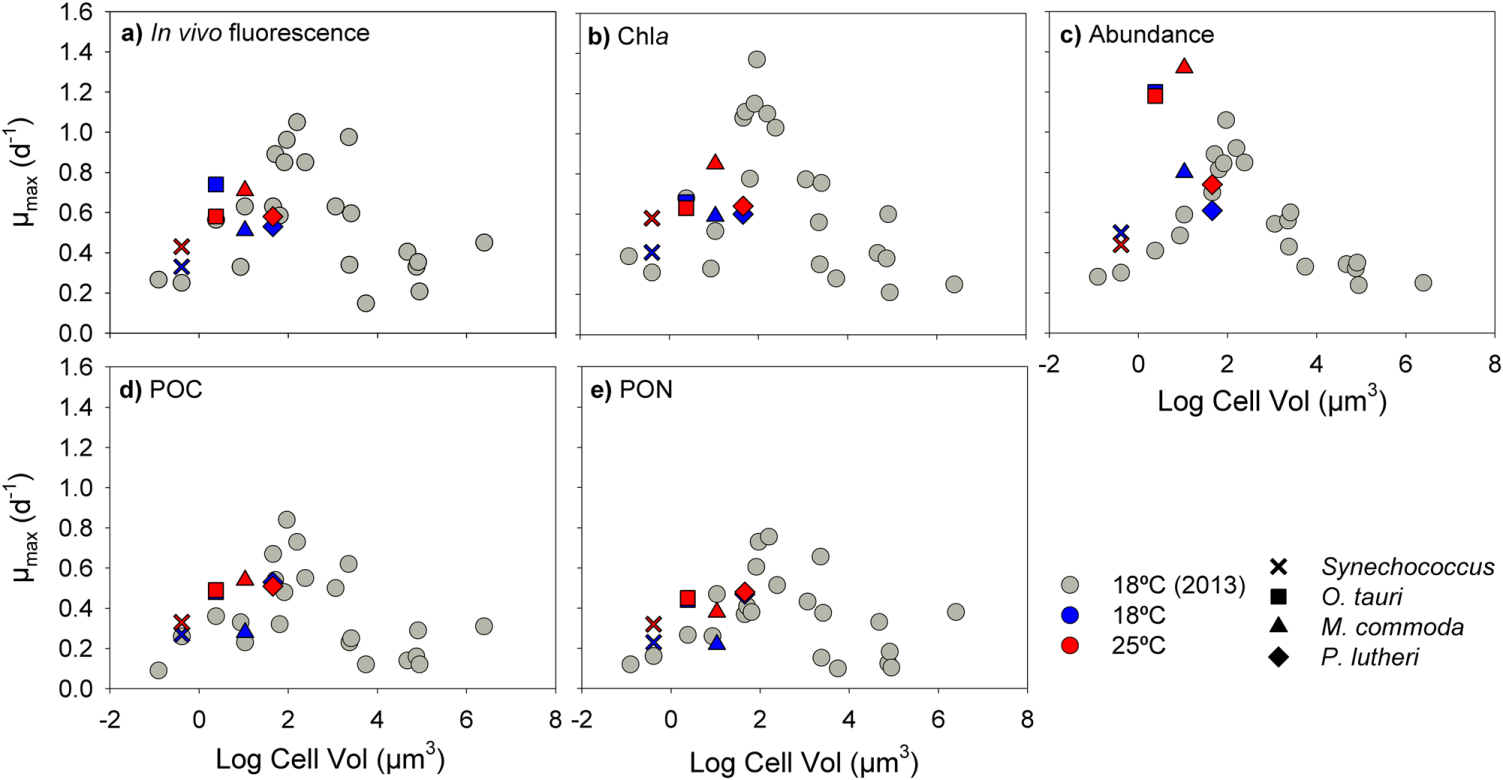
Relationship between cell volume (Vol , µm^3^) and maximum intrinsic growth rate (µ_max_, d^−1^) for the four species (*Synechococcus* sp., *Micromonas commoda*, *Ostreococcus tauri* and *Pavlova lutheri,* denoted by different symbols) grown at 18 °C (blue) and 25 °C (red). Also shown is the data for the 22 species grown at 18 °C (grey). All growth rates have been calculated with 5 metrics for measuring standing stocks (**a**) In vivo fluorescence, (**b**) chlorophyll *a* concentration (**c**) abundance, (**d**) POC concentration and e) PON concentration. Growth rate data at 18 °C based on cell abundance (panel **c**) are taken from Marañón et al.^9^, whereas all other growth rates based on additional metrics (panels **a**, **b**, **d** and **e**) are previously unreported data.

Maximum intrinsic growth rate (μ_max_) was significantly higher at 25 °C than at 18 °C for *M. commoda*, irrespective of the metric employed to assess phytoplankton stocks (Fig. 1, Table 1) (*t* test =  − 2.79, n = 47, *p* < 0.01). In the other three species, the differences in growth rates between temperatures were not significant. Comparing the current results of μ_max_ with those from the 2013 experiment (Marañón et al.^10^ and previous unreported data), growth rates were similar in both experiments at 18 °C (*t* test = 1.14, n = 114, *p* = 0.26).

**Table 1:** Maximum intrinsic growth rate (d^−1^) ± SE for the four species maintained at 18 and 25 °C. Growth rates were calculated with five metrics for measuring standing stocks (in vivo fluorescence, chlorophyll *a* concentration, abundance and particulate organic carbon and nitrogen content) are shown. The mean growth rate of successive growth cycles (n) is also given.

Specie	In vivo fluorescence			Chlorophyll *a*			Abundance			POC			PON		
	n	18 (°C)	25 (°C)	n	18 (°C)	25 (°C)	n	18 (°C)	25 (°C)	n	18 (°C)	25 (°C)	n	18 (°C)	25 (°C)
*Synechococcus*	3	0.33 ± 0.02	0.42 ± 0.03	3	0.41 ± 0.06	0.58 ± 0.09	3	0.50 ± 0.06	0.44 ± 0.06	1	0.27 ± 0	0.33 ± 0.04	1	0.23 ± 0	0.23 ± 0.03
*O. tauri*	3	0.74 ± 0.04	0.58 ± 0.02	3	0.66 ± 0.09	0.63 ± 0.03	2	1.2 ± 0.02	1.18 ± 0.14	1	0.49 ± 0.02	0.49 ± 0.03	1	0.44 ± 0.02	0.45 ± 0.02
*M. commoda*	3	0.51 ± 0.06	0.71 ± 0.04	3	0.59 ± 0.02	0.85 ± 0.07	2	0.8 ± 0.1	1.32 ± 0.24	3	0.28 ± 0.05	0.54 ± 0.14	3	0.22 ± 0.02	0.38 ± 0.03
*P. lutheri*	3	0.53 ± 0.02	0.58 ± 0.03	3	0.6 ± 0.02	0.64 ± 0.01	3	0.61 ± 0.06	0.74 ± 0.10	1	0.53 ± 0.01	0.51 ± 0.01	1	0.47 ± 0	0.48 ± 0

Discerning among the different metrics, we found significant or non-significant differences in growth rate between 18 and 25 °C depending on the metric used. This was the case of *O. tauri* whose differences in growth rate between the two temperatures were significant when in vivo fluorescence was used (*t* test = 3.75, n = 12, *p* < 0.01), but not when the other metrics were employed. Differences between temperatures were significant as well for *M. commoda* when using in vivo fluorescence (*t* test =  − 2.86, n = 11, *p* < 0.05), Chl*a* (*t* test =  − 3.72, n = 12, *p* < 0.01), POC (*t* test =  − 2.66, n = 10, *p* < 0.05) or PON (*t* test =  − 4.70, n = 10, *p* < 0.01) but not with cell abundance. For all the other species we did not find significant differences between 18 and 25 °C in any of the metrics used.

The absolute value of the calculated growth rate depended on the metric employed for measuring standing stocks. For example, *O. tauri* showed significantly higher values of µ_max_ (at both temperatures) based on cell abundance compared to the other metrics for measuring standing stocks (F(4,51) = 79, *p* < 0.01, Tuckey post-hoc test *p* < 0.01). Considering the data for the 22 species measured at 18 °C, μ_max_ calculated with the different metrics showed a strong correlation among them in all cases (R^2^ > 0.74, n = 22, *p* < 0.01) with the best correlation being found between POC and PON (R^2^ = 0.95, n = 22, *p* < 0.01). However, the absolute value of growth rate for the same species differed depending on the metric used. Our data showed that µ_max_ calculated with in vivo fluorescence, Chl*a* concentration and cell abundance tended to overestimate the biomass production rate (µ_max_ derived from POC) (Table 1, Fig S2) by 67 ± 13% for in vivo fluorescence, 97 ± 17% for Chl*a*, and 73 ± 14% for abundance (mean ± SE).

### Literature data analysis

**Figure 2:**
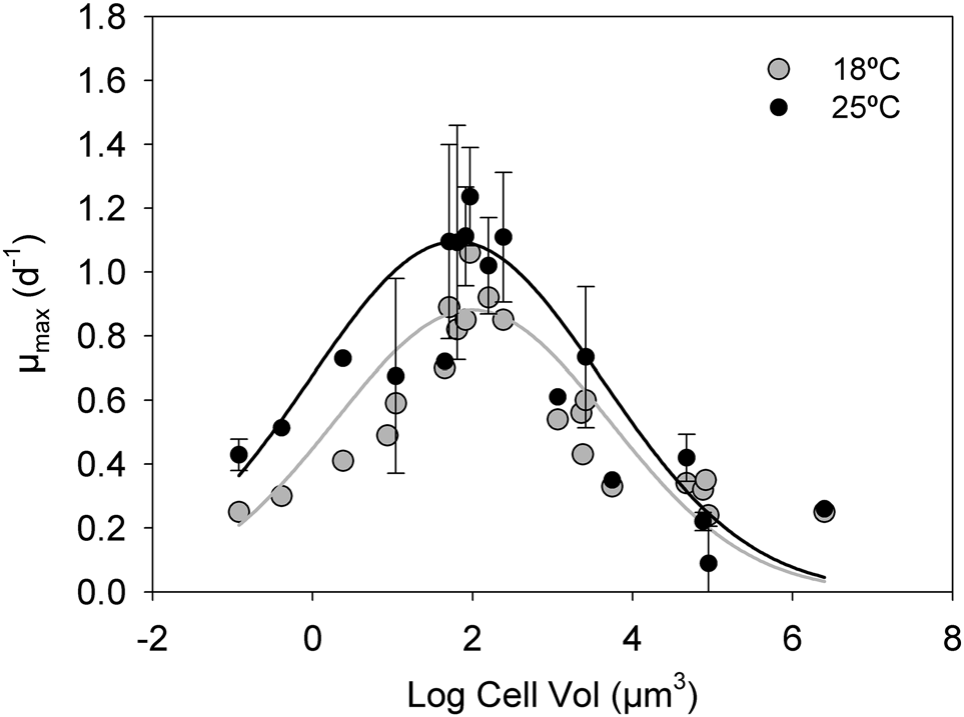
Unimodal relationship between growth rate (µ, d^−1^) and cell volume (Vol, µm^3^). Shown is the growth rate experimentally measured at 18 °C by Marañón et al.^10^ (grey dots) together with the estimated growth rate at 25 °C for the same species extracted from temperature-growth curves in the global data bases (black dots). Both curvatures were adjusted to a 3-parameter Gaussian model *f* = *a·exp(− 0.5·((x − x*_*0*_*)/b)*^2^*)*, where *a* is the height of the curve’s peak, *b* is the width and x_0_ is the position of the center). Grey line at 18 °C, a = 0.88, b = 1.72, x_0_ = 1.99 and R^2^ = 0.89, n = 22, *p* < 0.01; and black line at 25 °C, a = 1.09, b = 1.83, x_0_ = 1.79 and R^2^ = 0.69, n = 97, *p* < 0.01. For visualization purposes, when there were several estimates of growth rate at 25 °C for the same species, the mean was plotted. However, the fitting was done with all individual data points (see Figure S4). Error bars represent standard deviation.

Estimated μ_max_ at 25 °C predicted from the thermal growth curves of the different species and the original data of μ_max_ at 18 °C from the 2013 study^10^ were fitted to a Gaussian model to compare differences between temperatures (see parameters in the legend of Figs. 2 and S4). The results showed significant differences between the rates measured at 18 °C and at 25 °C (*t* test =  − 3.42, n = 119, *p* < 0.01). However, in spite of these differences, the unimodal pattern is observed also at 25 °C.

These unimodal patterns can be separated in two slopes, a positive slope below 300 µm^3^ of cell volume and a negative slope from 50 µm^3^ to the largest species. There was a positive slope for the first part of the curve at both 18 °C and 25 °C (slope = 0.22, R^2^ = 0.88, *p* < 0.01 and slope = 0.22, R^2^ = 0.63, *p* < 0.01, respectively). This shows that growth rate increases with cell size until approximately 300 µm^3^ of cell volume with no effect of temperature (F = 0.005, n = 117, *p* > 0.05).

For species larger than 50 µm^3^, the slope was negative at both temperatures (slope =  − 0.21, R^2^ = 0.89, *p* < 0.01 and slope =  − 0.27, R^2^ = 0.65, *p* < 0.01, for 18 and 25 °C, respectively). Again, the differences between the slopes at 18 and 25 °C were not significant (F = 0.00, n = 164, *p* > 0.05).

**Figure 3:**
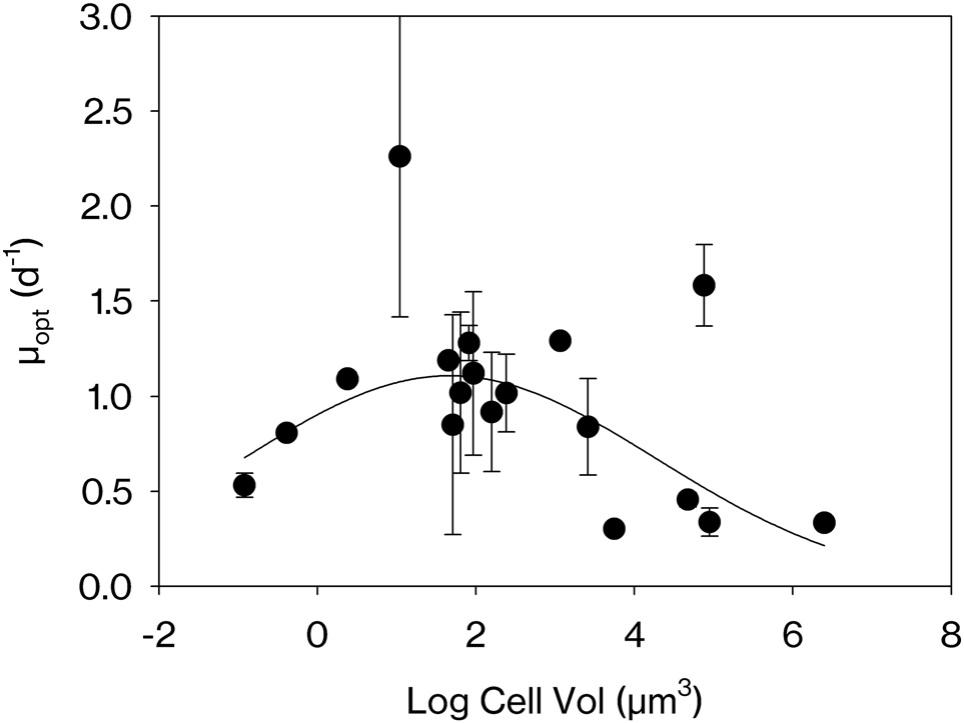
Relationship between the growth rate measured at the optimal temperature for growth (µ_opt_, d^−1^) and cell volume (Vol, µm^3^) extracted from the global databases. Data was adjusted to a 3-parameter Gaussian model *f* = *a*·exp(− 0.5·((*x* − *x*_0_)/*b*)^2^), where *a* is the height of the curve’s peak, *b* is the width and x_0_ is the position of the center. *a* = 1.10, *b* = 2.60, *x*_0_ = 1.67, and R^2^ = 0.22, n = 67, *p* < 0.01. For visualization purposes, when there were several estimates of growth rate for the same species, the mean was plotted. However, the fitting was done with all individual data points (see Figure S4). Error bars represent standard deviation.

Finally, we also calculated the growth rate at optimal temperature (µ_opt_) and we found that a significant unimodal pattern is also present in this data set (Fig. 3).

## Discussion

Our experimental measurements of maximum growth rates together with the analysis of thermal responses reported in the literature suggest that, although there is a slight effect of temperature on the relationship between phytoplankton cell size and growth rate, the unimodal size scaling pattern persists at 25 °C and at the optimal temperature for growth. This finding has implications for our understanding of the factors that control the biogeography of phytoplankton size structure in the ocean.

The positive relationship between growth rate and cell volume in small-sized species was maintained at both assayed temperatures. The µ_max_ measured in our laboratory experiments was higher at 25 °C than at 18 °C in three of the four species tested, irrespective of the metric employed (except *O. tauri*). However, these differences were only significant for *M. commoda*, and the effect of the warmer temperature was not sufficiently strong to force the species within the pico- and nanophytoplankton to grow as fast as those of larger cell size.

In general, the measured µ_max_ in the experiments was within the range of growth rates reported by Marañón et al.^10^ except for *O. tauri*, which showed significantly higher values than those previously reported, but only when cell abundance was used as a metric for measuring standing stocks. Chl*a* concentration, in vivo fluorescence and cell abundance are commonly used as a proxy of phytoplankton biomass. However, an underappreciated fact is that the absolute value of µ_max_ can be influenced by the type of measurement used to assess standing stocks. Our results show that these three metrics (in vivo fluorescence, Chl*a* and cell abundance) yield consistently higher values of µ_max_ than those obtained with biomass metrics (POC and PON), which reflects changes in resource allocation associated with unbalanced growth. Upon transfer to nutrient-rich medium in batch cultures, the population enters the exponential growth phase and cells allocate more resources into photosynthetic machinery^18,19^ while their cell size decreases as population growth proceeds fast^20,21^, in both cases leading to estimates of µ_max_ that exceed the actual rate of production of new biomass. Although the temperature dependence of growth rate is expected to be the same whatever metric is employed, the absolute value of growth rate is likely to differ depending on the metric used, which can affect some comparisons between studies.

We found significant differences between the growth rate measured at 18 °C and the estimated growth rate at 25 °C for the species used previously by Marañón et al.^10^. However, the relationship between cell size and growth was still unimodal at both temperatures. This result agrees with previous experimental^7–9^ and model-based^22^ studies which demonstrated that the mass-specific growth rate of intermediate-size cells can be higher than those of their smaller counterparts. This pattern may reflect the fact that the growth rate of the prokaryotes and the smallest eukaryotes is limited because of increased maintenance metabolic costs due to restricted cell space available relative to larger cells^23^. More recently, Stawiarski et al.^24^, in a laboratory experiment with 9 prokaryotic and eukaryotic picophytoplankton species spanning a range of 0.05–8 µm^3^ in cell volume (0.5–3 µm in ESD), showed that species of intermediate cell size grew faster than both smaller and larger ones, which resulted in a curved relationship between cell size and maximum growth rate, peaking at approximately 2 µm of ESD.

Other studies have not found a significant unimodal log–log relationship between growth rate and cell size at high temperatures (*e.g*. > 27°C^13^). However, temperatures above 27 °C are uncommon in the ocean and might be penalising species within the intermediate to large cell-size range, which usually have been isolated from temperate and polar regions. On the other hand, picophytoplankton have been shown to display a stronger temperature sensitivity than larger phytoplankton^12^ although the underlying mechanism is still unclear. This pattern might result not necessarily from cell size itself but from the biogeographical distribution of the different strains investigated, because the smallest phytoplankton species have often been isolated in tropical environments and are maintained at warmer temperature ranges. For example, the small species used in our experiment had been isolated in tropical and subtropical areas with sea surface temperatures higher than 19 °C (except *P. lutheri*, which had been isolated at 11 °C), which means that 25 °C was closer to their optimal temperature, thus explaining the differences found between the two temperatures tested. However, even though 18 °C was suboptimal for the growth of these species, we found no evidence to suggest that they were strongly limited by this experimental temperature^25–28^. Recently, Barton & Yvon-Durocher^29^, in an experimental study with 18 species, found that the relationship between cell size and growth rate at optimal temperature was best described by a negative linear log–log relationship with a size scaling exponent of − 0.088, whereas our results suggest a unimodal pattern also at the optimal temperature for each species. The different size-scaling patterns obtained in different studies may reflect, in part, differences in the individual species used as well as in the data coverage of the smallest cell sizes.

The unimodal pattern of phytoplankton growth could arise from phylogenetic differences along the cell size spectrum^13^. However, this would not explain why the unimodal pattern has been also observed within species of the same taxonomic group^7^. Large databases of phytoplankton growth rates allow to test if the tendency for intermediate-size species to reach the highest growth rates is encountered in different taxonomic groups. As shown by Kremer et al.^30^ (their Fig. 1) for cyanobacteria, green algae and diatoms, the highest growth rates (the upper limit of the data distribution) increase with increasing cell size up until a certain cell size and then decrease. This result suggests that the unimodal size-scaling of phytoplankton growth does not arise from phylogenetic differences along the size spectrum, although the latter undoubtedly contribute to the observed variability. Because the growth responses to temperature are strongly related to the temperature regime at the location where strains were isolated^15^, and considering that different taxa tend to be isolated from different regions (e.g. picocyanobacteria from warm regions, diatoms from temperate and polar regions), the association between phylogeny and thermal traits measured in the laboratory may not be necessarily causal. In fact, comparison of thermal response curves of different strains of the same species isolated from different locations reveals markedly distinct thermal traits^27,28^, with isolates from warmer locations exhibiting higher values of T_opt_.

The unimodal size scaling of phytoplankton maximum growth rates is relevant to understand the variability in community structure and size structure in natural phytoplankton assemblages. The left-hand side of the unimodal pattern predicts a positive relationship between cell size and growth rate within the picophytoplankton size range. A direct test of this prediction is provided by measurements of single-cell metabolic activity (isotope uptake rates) in *Prochlorococcus*, *Synechococcus* and picoeukaryotes. Working in the California Coastal Current, Berthelot et al.^31^ found that mean C-specific C fixation rates (equivalent to division rates, d^−1^) were higher in picoeukaryotes (1.7 µm in cell diameter, division rate of *ca*. 0.35 d^−1^) than in *Synechococcus* (1.2 µm, *ca*. 0.25 d^−1^), whose division rates in turn were higher than those of *Prochlorococcus* (0.6 µm, < 0.1 d^−1^). The same pattern was found with N-specific N uptake rates, which increased from *Prochlorococcus* (*ca*. 0.005 h^−1^) to *Synechococcus* (0.015 h^−1^) and then the picoeukaryotes (*ca*. 0.025 h^−1^). In a different study, conducted in the subtropical North Atlantic, Duhamel et al.^32^ reported that the mean biovolume-specific C fixation rate for *Prochlorococcus*, *Synechococcus* and the picoeukaryotes was 22 ± 4, 49 ± 17 and 101 ± 11 fgC µm^−3^ h^−1^, respectively, again demonstrating a positive relationship between cell size and growth rate. The increase in growth rates with cell size within the picophytoplankton size range has also been observed in experiments using the dilution technique. Worden et al.^33^ reported that mean growth rates for *Prochlorococcus*, *Synechococcus* and picoeukaryotes were 0.33, 0.52 and 0.71 d^−1^, respectively, in the Southern California Bight. More recently, Gutiérrez-Rodríguez et al.^34^ showed that picoeukaryotes sustained higher growth rates (0.9 d^−1^) than *Prochlorococcus* and *Synechococcus* (0.57–0.59 d^−1^) in the Costa Rica Dome. The positive relationship between cell size and growth rate was also reported for picocyanobacteria in the California Current^35^. More broadly, the trend of increasing mass-specific metabolic rate with increasing cell size in prokaryotes has been found both in global data analyses^36^ and trans-oceanic latitudinal cruises^37^.

Near-maximum growth rates are attained in the field under nutrient-sufficient conditions and therefore the left-hand size of the unimodal pattern predicts increasing growth rate and dominance of progressively larger cells as resource availability increases. However, the connection between group-specific growth rates and community structure is not immediate because trophic interactions such as grazing are also relevant. Thus, large cells may dominate phytoplankton biomass during blooms not because they grow faster but because they suffer lower grazing pressure than their smaller counterparts^38^. However, over broad biogeographic scales in the ocean, regions of increased phytoplankton biomass do show faster phytoplankton growth rates^39,40^**.** At the level of functional groups, there is also evidence that diatoms sustain faster growth rates in conditions when they contribute most to the community biomass^41–43^. In this connection, the association between a higher ability to sustain fast growth rates and an enhanced contribution to total biomass agrees with the observation that picoeukaryotes tend to become dominant over the picocyanobacteria *Synechococcus* and *Prochlorococcus* as conditions become less oligotrophic^31,44,45^. A similar result has been described also for phytoplankton of larger cell sizes, whereby the size distribution of pico- and nanoeukaryotes in the Atlantic Ocean shifted from a biomass dominance of picoeukaryotes in oligotrophic regions towards a dominance by nanoeukaryotes in more nutrient-rich waters^46^.

Finally, the unimodal size scaling of phytoplankton growth implies that the species of intermediate cell size can be expected to dominate under conditions of high resource supply. While measurements of size-fractionated Chl*a* concentration indicate that the microphytoplankton dominate when total phytoplankton biomass is high^47,48^, this result likely arises from the fact that bloom-forming species such as diatoms tend to form chains that are trapped by the 20-µm filter even if individual cell diameter is smaller than 20 µm. In fact, biovolume data based on flow cytometry and microscopy show that intermediate-size cells (nanophytoplankton) are the dominant size class during bloom events in the NW Iberian upwelling region^2^ and in eutrophic coastal lagoons characterized by large phytoplankton biomass^49^.

## Conclusions

We have shown that the unimodal relationship between phytoplankton cell size and growth rate, previously reported at 18 °C, persists at 25 °C and under optimal growth temperature. These results add to the growing evidence indicating that Kleiber’s rule is not applicable to photosynthetic unicells when the entire cell size range is considered. The unimodal size scaling pattern is supported by independent field observations indicating that 1) phytoplankton metabolic rates increase with cell size in the picophytoplankton size range and 2) cells of intermediate cell size dominate during conditions on increased resource supply, when fast-growing species are favoured.

## Methods

### Experimental design

We grew four species of phytoplankton with cell sizes spanning the range 0.41–45 µm^3^ in cell volume (Table S1), which corresponds to the left-hand side of the unimodal pattern of size scaling of growth rate reported by Marañón et al.^10^. We maintained cultures at 18 °C and at 25 °C to assess the effect of temperature on the left-hand side slope of the cell size-growth rate relationship. The species considered were *Synechococcus* sp. (RCC33), *Ostreococcus tauri* (RCC745, previously known as RCC116), *Micromonas commoda* (RCC496) and *Pavlova lutheri* (RCC1537, recently named *Diachronema lutheri*). Cultures were grown in duplicate 250-ml flasks and growth conditions were identical to those used by Marañón et al.^10^ (Table S1). Irradiance was set at 250 µmol photons m^−2^ s^−1^ with a 12:12 L:D photoperiod. Cultures were acclimated at a given temperature for at least 10 generations. During this period, we took samples every 2 days to determine in vivo fluorescence and photosystem II quantum yield (F_v_/F_m_). After the acclimation period, cultures were allowed to complete 3 successive growth cycles, from inoculation to late exponential phase, during which we took daily samples for in vivo fluorescence, F_v_/F_m_, chlorophyll *a* content, and abundance. During the second growth cycle, we also took daily samples for particulate organic carbon and nitrogen content (POC and PON).

### Measurement of standing stocks

Phytoplankton standing stocks were measured with five metrics: in vivo fluorescence, Chl*a* concentration, cell abundance and particulate organic carbon and nitrogen (POC and PON). In vivo fluorescence was measured with a Turner Aquafluor portable fluorometer (Turner Designs, CA, USA). Chlorophyll *a* (Chl*a*) concentration was measured fluorometrically on a TD-700 Turner Designs fluorometer (Turner Designs, CA, USA) after filtration of 5-mL samples onto GF/F filters, which were frozen at − 20 °C and extracted with 90% HPLC-grade acetone. For the determination of cell abundance, 1.8-mL samples were taken, fixed with 180 µL of a solution of paraformaldehyde and glutaraldehyde (10%) and fast-frozen in liquid nitrogen until analysis in a BD FACSCalibur flow cytometer (BD Biosciences, CA, USA). For POC and PON determination, 10–20 mL samples were filtered through pre-combusted GF/F filters and stored at − 20 °C. Prior to analysis, filters were desiccated at room temperature for 48 h and samples were analysed using a Carlo Erba Instruments EA1108 elemental analyser (CE Instruments Ltd, Wigan, UK).

### Calculation of growth rates

The maximum growth rate (µ_max_) was determined for each species and temperature as the slope of the linear regression between time (days) and the natural logarithm of each metric of standing stock (abundance, Chl*a* concentration, in vivo fluorescence, POC and PON concentration) during the exponential growth phase. µ is reported as the mean value ± SD of the successive growth cycles on each replicate culture performed for each species at each temperature.

### Literature data analysis

We used data sets from the literature compiled by Bissinger et al.^14^, Chen et al.^15^, Thomas et al.^16^, and Heinle^17^ (data available in https://doi.org/10.17882/75434). These databases contain measurements of maximum growth rate at different temperatures for 18 of the 22 different species and strains used by Marañón et al.^10^. We only used data from studies that (i) included at least 3 temperatures between 18 and 25 °C and (ii) were conducted under experimental conditions similar to those used by Marañón et al.^10^. The latter include nutrient-replete conditions, a light:dark photoperiod of 12L:12D or 14L:10D and PAR higher than 50 µmol photons m^−2^ s^−1^ .We were not able to find experimental data with these conditions for *Nannochloropsis gaditana*, *Melosira nummuloides*, *Protoceratium reticulatum* and *Coscinodiscus radiatus.*

In 43% of all thermal response curves found, the number of growth rate determinations at different temperatures was 6 or less. In these cases, we fit the growth rate into a linear equation (following Chen and Laws^50^). This fit was done in R statistical software (v. 3.5.0) using the ‘lm’ function from ‘stats’ package (Eq. 1). The goodness of fit was calculated with the coefficient of determination R^2^.

In the rest of the cases (57%), where the number of observations of growth rate versus temperature was larger than 6, the data were fit to linear (Eq. 1), exponential (Eq. 2^51^), and non-linear optimum models (Eq. 3^52,53^):

1 $$\upmu _{\max } = {\text{a }} + {\text{ b}} \times {\text{T}}$$

2 $$\upmu _{\max } = {\text{a }} + {\text{ b}}^{{\text{T}}}$$

3 $$\upmu _{\max } = c e\frac{ - E}{{KT}} /1 + e \frac{ - 1}{{KT}}\left( {E_{{\text{D}}} - \left( {\frac{{E_{{\text{D}}} }}{{T_{opt} }} + K\ln \left( {\frac{E}{{E_{{\text{D}}} - E}}} \right)} \right)T} \right)$$

where μ_max_ is the maximum growth rate at a certain temperature, T is the temperature in Celsius degrees in Eqs. (1 and 2) and in Kelvin degrees in Eq. (3), T_opt_ is the optimum temperature, K is the Boltzmann’s constant, and E and E_D_ indicate the steepness of the increase or decline, respectively, of μ_max_ at temperatures below or above T_opt_.

Data fitting to the models described by Eqs. (2 and 3) was conducted using the package ‘nls.multstart’ in R statistical software (v. 3.5.0) with a maximum of 100,000 iterations and reasonable initial values to improve algorithm convergence. The goodness of fit for each of these functions was estimated by using the Akaike’s criteria (AIC) corrected for small number of observations (AIC_C_):

4 $${\text{AIC}}_{{\text{C}}} = {\text{ AIC }} + \, \left( {{\text{2K }}* \, \left( {{\text{K}} + {1}} \right) \, /{\text{ n}} - {\text{K}} - {1}} \right)$$

where K is the number of parameters covered by the function plus 1 and n is the number of observations. In this way we obtained empirical functions that relate temperature to growth rate, and for each strain and experiment we selected the function with the best fit as indicated the smallest value of AIC_C_. We then used the growth rate measured at 18 °C by Marañón et al.^10^ to correct the model’s intercept, thus representing the effect on growth of the experimental conditions used in 2013, and this new temperature-growth function allowed us to estimate µ at 25 °C.

When the available data allowed it, we also estimated µ_opt_ (measured at T_opt_) by applying Eq. (3). For growth curves with less than 6 data points we checked data individually to select whether µ_opt_ had been reached at T_opt_. Overall, we obtained 94 estimates of growth rate at 25 °C and at the maximum/optimal temperature for growth based on 766 temperature-growth rate data points measured in 18 species and reported in 30 publications.

### Statistical analyses

To test for significant differences in the maximum growth rates of *Synechococcus* sp. (RCC33), *Ostreococcus tauri* (RCC745), *Micromonas commoda* (RCC496) and *Pavlova lutheri* (RCC1537) determined at 18 and 25 °C with data of in vivo fluorescence, Chl*a*, abundance, POC and PON we conducted a parametrical Student´s *t* test. To check for significant differences in the maximum growth rate between 18 °C (from Marañón et al.^10^) and 25 °C (obtained from the literature data analysis) we performed the same test and fit data to a three-parameter Gaussian curve in Sigma Plot V.10.0 (SysTat Software, Berkshire, UK). Regression analysis of data within the increasing (left-hand side) and decreasing (right-hand side) part of the cell size-growth rate relationship was also done and regression coefficients were compared by ANCOVA analysis. Three-parameter Gaussian curves were fit as well for the relationship between optimum growth rates and cell volume. All data were checked with a homoscedasticity Levene’s test as well as with a normality Shapiro–Wilks’s test. All statistical analyses were done in IBM SPSS Statistics v.22 (IBM Corp., Armonk, N.Y., USA) and R Studio (R statistical software, Boston, Massachusetts, US).

## Supplementary information

Supplementary Information.

## References

[1] Finkel ZV (2010). Phytoplankton in a changing world: cell size and elemental stoichiometry. J. Plankton Res..

[2] Marañón E (2015). Cell size as a key determinant of phytoplankton metabolism and community structure. Ann. Rev. Mar. Sci..

[3] Chavez FP, Messié M, Pennington JT (2011). marine primary production in relation to climate variability and change. Ann. Rev. Mar. Sci..

[4] Kleiber M (1932). Body size and metabolism. Hilgardia J. Agric. Sci..

[5] Gillooly JF (2001). Effects of size and temperature on metabolic rate. Science.

[6] Brown JH, Gillooly JF, Allen AP, Savage VM, West GB (2004). Toward a metabolic theory of ecology. Ecology.

[7] Raven JA (1994). Why are there no picoplanktonic O^2^ evolvers with volumes less than 10^–19^ m^3^?. J. Plankton Res..

[8] Bec B, Collos Y, Vaquer A, Mouillot D, Souchu P (2008). Growth rate peaks at intermediate cell size in marine photosynthetic picoeukaryotes. Limnol. Oceanogr..

[9] Chen B, Liu H (2010). Relationships between phytoplankton growth and cell size in surface oceans: interactive effects of temperature, nutrients, and grazing. Limnol. Oceanogr..

[10] Marañón E (2013). Unimodal size scaling of phytoplankton growth and the size dependence of nutrient uptake and use. Ecol. Lett..

[11] Ward BA, Marañón E, Sauterey B, Rault J, Claessen D (2016). The size dependence of phytoplankton growth rates: a trade-off between nutrient uptake and metabolism. Am. Nat..

[12] Chen B, Liu H, Huang B, Wang J (2014). Temperature effects on the growth rate of marine picoplankton. Mar. Ecol. Prog. Ser..

[13] Sal S, Alonso-Saez L, Bueno J, Garcıa FC, Lopez-Urrutia A (2015). Thermal adaptation, phylogeny, and the unimodal size scaling of marine phytoplankton growth. Limnol. Oceanogr..

[14] Bissinger JE, Montagnes DJS, Sharples J, Atkinson D (2008). Predicting marine phytoplankton maximum growth rates from temperature: improving on the Eppley curve using quantile regression. Limnol. Oceanogr..

[15] Chen B (2015). Patterns of thermal limits of phytoplankton. J. Plankton Res..

[16] Thomas MK, Kremer CT, Litchman E (2016). Environment and evolutionary history determine the global biogeography of phytoplankton temperature traits. Glob. Ecol. Biogeogr..

[17] Heinle, M. The effects of light, temperature and nutrients on coccolithophores and implications for biogeochemical models (Doctoral dissertation, University of East Anglia, Norwich, United Kingdom). (2013).

[18] Kruskopf M, Flynn KJ (2006). Chlorophyll content and fluorescence responses cannot be used to gauge reliably phytoplankton biomass, nutrient status or growth rate. New Phytol..

[19] Flynn KJ, Raven JA (2016). What is the limit for photoautotrophic plankton growth rates?. J. Plankton Res..

[20] Prakash A, Skoglund L, Rystad B, Jensen A (1973). Growth and cell-size distribution of marine planktonic algae in batch and dialysis cultures. J. Fish. Res. Board Canada.

[21] Xia L, Huang R, Li Y, Song S (2017). The effect of growth phase on the surface properties of three oleaginous microalgae (*Botryococcus* sp. FACGB-762, *Chlorella* sp. XJ-445 and *Desmodesmus bijugatus* XJ-231). PLoS ONE.

[22] Verdy A, Follows M, Flierl G (2009). Optimal phytoplankton cell size in an allometric model. Mar. Ecol. Prog. Ser..

[23] Kempes CP, Dutkiewicz S, Follows MJ (2012). Growth, metabolic partitioning, and the size of microorganisms. Proc. Natl. Acad. Sci. U.S.A..

[24] Stawiarski B, Buitenhuis ET, Quéré CL (2016). The physiological response of picophytoplankton to temperature and its model representation. Front. Mar. Sci..

[25] Martiny AC, Ma L, Mouginot C, Chandler JW, Zinser ER (2016). Interactions between thermal acclimation, growth rate, and phylogeny influence prochlorococcus elemental stoichiometry. PLoS ONE.

[26] Mackey KRM (2013). Effect of temperature on photosynthesis and growth in marine *Synechococcus* spp. Plant Physiol..

[27] Demory D (2018). Picoeukaryotes of the *Micromonas* genus: sentinels of a warming ocean. ISME J..

[28] Pittera J (2014). Connecting thermal physiology and latitudinal niche partitioning in marine *Synechococcus*. ISME J..

[29] Barton S, Yvon-Durocher G (2019). Quantifying the temperature dependence of growth rate in marine phytoplankton within and across species. Limnol. Oceanogr..

[30] Kremer CT, Thomas MK, Litchman E (2017). Temperature- and size-scaling of phytoplankton population growth rates: reconciling the Eppley curve and the metabolic theory of ecology. Limnol. Oceanogr..

[31] Berthelot H (2019). NanoSIMS single cell analyses reveal the contrasting nitrogen sources for small phytoplankton. ISME J..

[32] Duhamel S, Kim E, Sprung B, Anderson OR (2019). Small pigmented eukaryotes play a major role in carbon cycling in the P-depleted western subtropical North Atlantic, which may be supported by mixotrophy. Limnol. Oceanogr..

[33] Worden AZ, Nolan JK, Palenik B (2004). Assessing the dynamics and ecology of marine picophytoplankton: the importance of the eukaryotic component. Limnol. Oceanogr..

[34] Gutierrez-Rodríguez A, Selph KE, Landry MR (2015). Phytoplankton growth and microzooplankton grazing dynamics across vertical environmental gradients determined by transplant in situ dilution experiments. J. Plankton Res..

[35] Worden AZ, Binder BJ (2003). Application of dilution experiments for measuring growth and mortality rates among *Prochlorococcus* and *Synechococcus* populations in oligotrophic environments. Aquat. Microb. Ecol..

[36] DeLong JP, Okie JG, Moses ME, Sibly RM, Brown JH (2010). Shifts in metabolic scaling, production, and efficiency across major evolutionary transitions of life. Proc. Natl. Acad. Sci. U.S. A..

[37] García FC (2016). The allometry of the smallest: superlinear scaling of microbial metabolic rates in the Atlantic Ocean. ISME J..

[38] Kiørboe T (1993). Turbulence, phytoplankton cell size, and the structure of pelagic food webs. Adv. Mar. Biol..

[39] Marãnón E (2014). Resource supply overrides temperature as a controlling factor of marine phytoplankton growth. PLoS ONE.

[40] Behrenfeld MJ, Boss E, Siegel DA, Shea DM (2005). Carbon-based ocean productivity and phytoplankton physiology from space. Global Biogeochem. Cycles.

[41] Tsuda A (2003). A mesoscale iron enrichment in the Western subarctic Pacific induces a large centric diatom bloom. Science.

[42] Latasa M, Landry MR, Schlüter L, Bidigare RR (1997). Pigment-specific growth and grazing rates of phytoplankton in the central equatorial pacific. Limnol. Oceanogr..

[43] Cavender-Bares KK, Mann EL, Chisholm SW, Ondrusek ME, Bidigare RR (1999). Differential response of equatorial Pacific phytoplankton to iron fertilization. Limnol. Oceanogr..

[44] Mouriño-Carballido B (2016). Nutrient supply controls picoplankton community structure during three contrasting seasons in the northwestern Mediterranean Sea. Mar. Ecol. Prog. Ser..

[45] Schmidt K (2020). Increasing picocyanobacteria success in shelf waters contributes to long-term food web degradation. Glob. Chang. Biol..

[46] Tarran GA, Heywood JL, Zubkov MV (2006). Latitudinal changes in the standing stocks of nano- and picoeukaryotic phytoplankton in the Atlantic Ocean. Deep Res. Part II Top. Stud. Oceanogr..

[47] Marañón E, Cermeño P, Latasa M, Tadonléké RD (2012). Temperature, resources, and phytoplankton size structure in the ocean. Limnol. Oceanogr..

[48] Chisholm SW (1992). Phytoplankton Size. Prim. Product. Biogeochem. Cycles Sea.

[49] Montes-Pérez JJ (2020). Intermediate-size cell dominance in the phytoplankton community of an eutrophic, estuarine ecosystem (Guadalhorce River, Southern Spain). Hydrobiologia.

[50] Chen B, Laws EA (2016). Is there a difference of temperature sensitivity between marine phytoplankton and heterotrophs?. Limnol. Oceanogr..

[51] Eppley RW (1972). Temperature and phytoplankton growth in the sea. Fish. Bull..

[52] Johnson F, Lewin I (1946). The growth rate of *E. coli* in relation to temperature, Quinine and Coenzyme. J. Cell Physiol..

[53] Dell AI, Pawar S, Savage VM (2011). Systematic variation in the temperature dependence of physiological and ecological traits. Proc. Natl. Acad. Sci. U.S.A..

