# Supplementary material for: Effect of temperature on the unimodal size scaling of phytoplankton growth
Source: Sci Rep. 2021 Jan 13;11:953. doi: 10.1038/s41598-020-79616-0 (PMC7806832; doi:10.1038/s41598-020-79616-0)
Supplement: Supplementary file 1 — Supplementary Information. [file 41598_2020_79616_MOESM1_ESM.docx]

**Supplementary information**

**“Effect of temperature on the unimodal size scaling of phytoplankton growth”**

**Fernández-González, Cristina*^1,2^; Marañón, Emilio.^1,2^**

^1^Department of Ecology and Animal Biology, Universidade de Vigo, Spain

^2^Centro de Investigación Mariña (CIM-UVigo), Vigo, Spain

*c.fernandez@uvigo.es

Figure S1. Evolution of a) *In vivo* fluorescence, b) Chl*a* concentration, c) cell abundance, d) POC content and e) PON content in a growth cycle of *Synechococcus* sp., *Ostreococcus tauri*, *Micromonas commoda* and *Pavlova lutheri* growing at 18ºC (dashed lines) and 25ºC (continuous lines). Each dot represents the average of two replicates and error bars show standard deviation.

Figure S2. Comparison of the maximum intrinsic growth rate (µ_max_, d^-1^) calculated with the five different metrics for measuring standing stocks used. The black line represents the theoretical 1:1 relationship between µ_max_ calculated with a) *in vivo* fluorescence, b) Chl*a* concentration, c) cell abundance and d) PON with respect to the µ_max_ calculated as biomass (µ_max_ POC). Each dot represents one of the 22 species employed by Marañón et al.^9^ at 18ºC.

Figure S3. Maximum intrinsic growth rate (µ_max_, d^-1^) for the four species measured at 18ºC (grey symbols) and at 25ºC (black symbols) with five metrics for measuring standing stocks i*n vivo* fluorescence, Chl*a* concentration, cell abundance, POC and PON concentration).

Figure S4. Relationship between maximum growth rate (µ_max_, d^-1^) and cell volume (µm^3^). Represented are growth rate experimentally measured at 18ºC by Marañón et al.^9^ (grey dots) together with the estimated growth rate at 25ºC for the same species extracted from temperature-growth curves in the global data bases (black dots). Both curvatures were adjusted to a 3-parameter Gaussian model (*f*=*a*·exp(-0.5·((*x*-*x*_0_)/*b*)^2^)), where *a* is the height of the curve’s peak, *b* is the width and *x*_0_ is the position of the center). Grey line at 18˚C, a=0.88, b=1.72, x_0_=1.99 and R^2^=0.89, n=22, p<0.01; and black line at 25˚C, a=1.09, b=1.83, x_0_=1.79 and R^2^=0.69, n=97, p<0.01.

Figure S5. Relationship between the maximum growth rate measured at the optimal temperature for growth (µ_opt_, d^-1^) and cell volume (Vol in µm^3^) extracted from the global databases. Data was adjusted to a 3-parameter Gaussian model (*f*=*a*·exp(-0.5·((*x*-*x*_0_)/*b*)^2^)), where *a* is the height of the curve’s peak, *b* is the width and *x*_0_ is the position of the center). *a*=1.10, *b*=2.60, *x*_0_=1.67, and R^2^=0.22, n=67, p<0.01.

Table S1. Cell size represented by volume (µm^3^) and experimental growth conditions for each species. Cell volume was measured at 18ºC only.

|  | Volume (µm^3^ cell^-1^) | Temperature (⁰C) | | Daylength (L:D) | Irradiance (µmol photons m^-2^ s^-1^) | Culture media |
| --- | --- | --- | --- | --- | --- | --- |
|  |  |  |  |  |  |  |
| *Synechococcus sp.* | 0.41 | 18 | 25 | 12:12 | 250 | F4 |
| *O. tauri* | 2.4 | 18 | 25 | 12:12 | 250 | K2 |
| *M. commoda* | 10.7 | 18 | 25 | 12:12 | 250 | K2 |
| *Pavlova lutheri* | 45.4 | 18 | 25 | 12:12 | 250 | F4 |
